# Long-term durable response to Sintilimab therapy in synchronous lung squamous cell carcinoma and gastric adenocarcinoma: a rare case report

**DOI:** 10.3389/fonc.2026.1679799

**Published:** 2026-02-06

**Authors:** Margaret Mekuriya Bassaye, Zou Jijian, Zhou Li, Shuai Han

**Affiliations:** 1 Department of General Surgery, Zhujiang Hospital, Southern Medical University, Guangzhou, Guangdong, China; 2 General Surgery I, Weihaiwei People’s Hospital, Weihai, China

**Keywords:** gastric adenocarcinoma, immune checkpoint inhibitor, lung squamous cell carcinoma, Sintilimab, synchronous primary tumors, targeted therapy

## Abstract

Gastric cancer (GC) and lung squamous cell carcinoma (SCC) are major global causes of cancer-related morbidity and mortality, but their synchronous occurrence is exceedingly rare and poses substantial diagnostic and therapeutic challenges. This report describes a 64-year-old male with synchronous lung SCC and gastric adenocarcinoma who achieved long-term disease control with Sintilimab monotherapy. Initial imaging and biopsy confirmed poorly differentiated lung SCC (PD-L1 CPS: 2), while PET-CT and endoscopy identified a gastric lesion later confirmed as adenocarcinoma (PD-L1 CPS: 5). Despite indications for chemotherapy, the patient declined cytotoxic therapy and initially refused surgery. He was treated with Sintilimab plus Anlotinib, resulting in lung lesion regression. Progression of gastric obstruction led to gastrectomy two years later; subsequent lung recurrence was managed surgically. Both tumors showed high proliferative indices (Ki-67 ~80–90%) and PD-L1 expression, supporting responsiveness to immune checkpoint blockade. This case highlights the feasibility of immune monotherapy in managing complex synchronous malignancies, especially when standard treatments are declined.

## 1 Introduction

Gastric cancer (GC) and lung squamous cell carcinoma (SCC) are each among the leading causes of cancer morbidity and mortality globally (1–5). Individually, they are prevalent, but the synchronous presentation of the two distinct cancers in one patient is an extremely rare and clinically complex occurrence. Korean and Japanese population-based studies have approximated the prevalence of synchronous lung–gastric malignancies to be around 0.7%, while gastric cancer patients can have synchronous neoplasms in 3–10% of patients (6).

Synchronous primary cancers pose significant obstacles in clinical management due to overlapping symptoms (7), diagnostic uncertainty (8), and complex therapeutic decision-making (9, 10). These challenges become even more enhanced when a second malignancy is discovered incidentally during treatment of the first (9, 11). These overlapping clinical phenomena can result in delayed detection and treatment complexity, with potential impact on patient prognosis and survival (9). It is necessary to discriminate between synchronous primary tumors and metastatic disease for accurate prognosis and treatment plans, particularly in cases where synchronous tumors could require different strategies, because of origin, nature and stage of cancer (6).

While synchronous presentation of stomach cancer and lung squamous cell carcinoma remains rare, pulmonary metastasis (PM) of gastric cancer is of significant prognostic importance. PM from gastric cancer is rare in clinical practice, reported in 0.5–16% of GC patients with distant metastases, although postmortem studies reveal a higher incidence of 22–52%, suggesting many cases remain undetected during life. PMs were identified in approximately 5.56% of patients with an overall survival of only 2 months vs. 14 months without lung metastasis (5). According to Sun et al. (1) PM occurs in a subset of patients with newly diagnosed gastric cancer and are associated with reduced survival outcomes, underscoring their prognostic significance (1). This is in line with another report of the limited prevalence and challenging management of synchronous oligo-metastases including pulmonary ones, from gastric origin (12).

The independent risk factors for PM identified included, squamous cell carcinoma histology, late stage of the tumor (T4), metastasis in the nodes, and liver. The data indicated chemotherapy to be associated with more advanced diseases yet it improved outcomes (13). These findings highlight the clinical challenges in managing patients with synchronous or metastatic gastric and lung cancers. It also establishes the critical need for innovative therapies like immune checkpoint inhibitors.

The advent of immune checkpoint inhibitors (ICIs) has revolutionized oncology, offering superior survival in cancer of limited therapeutic availability, like metastatic melanoma or non-small cell lung cancer (14). ICIs improve immune recognition and elimination of cancer cells and have been found to have significant benefits in cancers like both lung SCC and gastric adenocarcinoma (14, 15).

Anti–PD-1/PD-L1 therapy (e.g. nivolumab and pembrolizumab, and atezolizumab and durvalumab) are approved in secondary lines of GCs, whereas ICIs are on-label standard of care in advanced non-small cell lung cancer, including squamous histology. Sintilimab, a human monoclonal antibody against PD-1, has proven to have positive antitumor activity with a favorable safety profile in both long-term NSCLC and GC settings (16, 17). In combination regimens, particularly in advanced or inoperable tumors, Sintilimab has also been co-administered with multi-targeted tyrosine kinase inhibitors (TKIs) such as Anlotinib, which blocks a variety of different pathways to inhibit angiogenesis and tumor proliferation synergistically with immune checkpoint blockade (18–20).

Despite considerable advances in immunotherapy, synchronous primary tumors of multiple histologies are rarely treated with immune checkpoint inhibitor (ICI) monotherapy due to the absence of strong guideline recommendations and limited clinical evidence. A case report by Ghammem et al. (21) highlights the infrequency of ICI use in multiple synchronous cancers, noting that clinical guidelines lack structured recommendations for such cases (21). This lack of evidence is also acknowledged in the practice guidelines of the Society for Immunotherapy of Cancer (SITC). For instance, the melanoma-specific guideline states that data on ICI monotherapy in complex cases involving synchronous disease are limited, recommending combination therapies only if supported by favorable evidence (22). Similarly, Greten et al. (23) emphasize that although ICI monotherapy has proven effective in single indications (e.g., HCC), its use in more complex or synchronous oncologic cases remains insufficiently validated for broad clinical endorsement. The absence of definitive outcomes data continues to impede standardization (23).

This case report uniquely documents successful long-term administration of Sintilimab monotherapy in a patient with synchronous lung SCC and gastric adenocarcinoma. Remarkably, durable disease control was achieved despite the patient’s refusal of conventional chemotherapy and initial reluctance toward surgery. This robust response supports the potential role of ICIs as monotherapy for managing multiple synchronous neoplasms, highlighting the importance of individualized treatment approaches and patient-centered care. The report details the entire clinical course, treatment regimen, imaging, histopathologic findings, and outcomes, contributing valuable insights into the feasibility and efficacy of immunotherapy in complex oncologic scenarios involving synchronous primary tumors.

## 2 Case description

**Figure 1:**
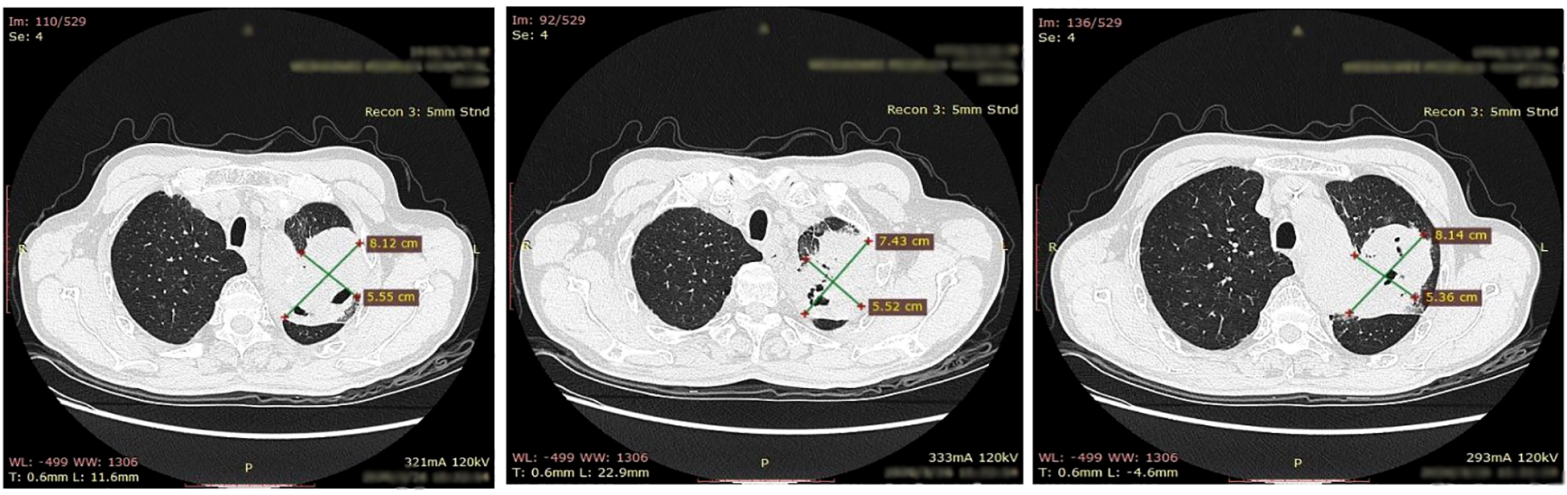
Axial CT images of the upper chest demonstrating an irregular lesion in the left upper lung lobe.

A 64-year-old male with a medical history of diabetes mellitus, hypertension, and chronic hepatitis B underwent a chest computed tomography (CT) scan at an outside hospital on March 26, 2020. The axial slices from the upper chest region revealed a measurable lesion in the upper lobe of the left lung. The lesion appeared as an irregular mass with maximum dimensions of approximately 7.4–8.1 cm by 5.4–5.6 cm in the one and perpendicular axial diameters, respectively. Areas of cavitation or necrosis were also noted within the lesion. Additionally, the CT scan suggested suspected metastases to the hilar and mediastinal lymph nodes (**Figure 1**).

**Figure 2:**
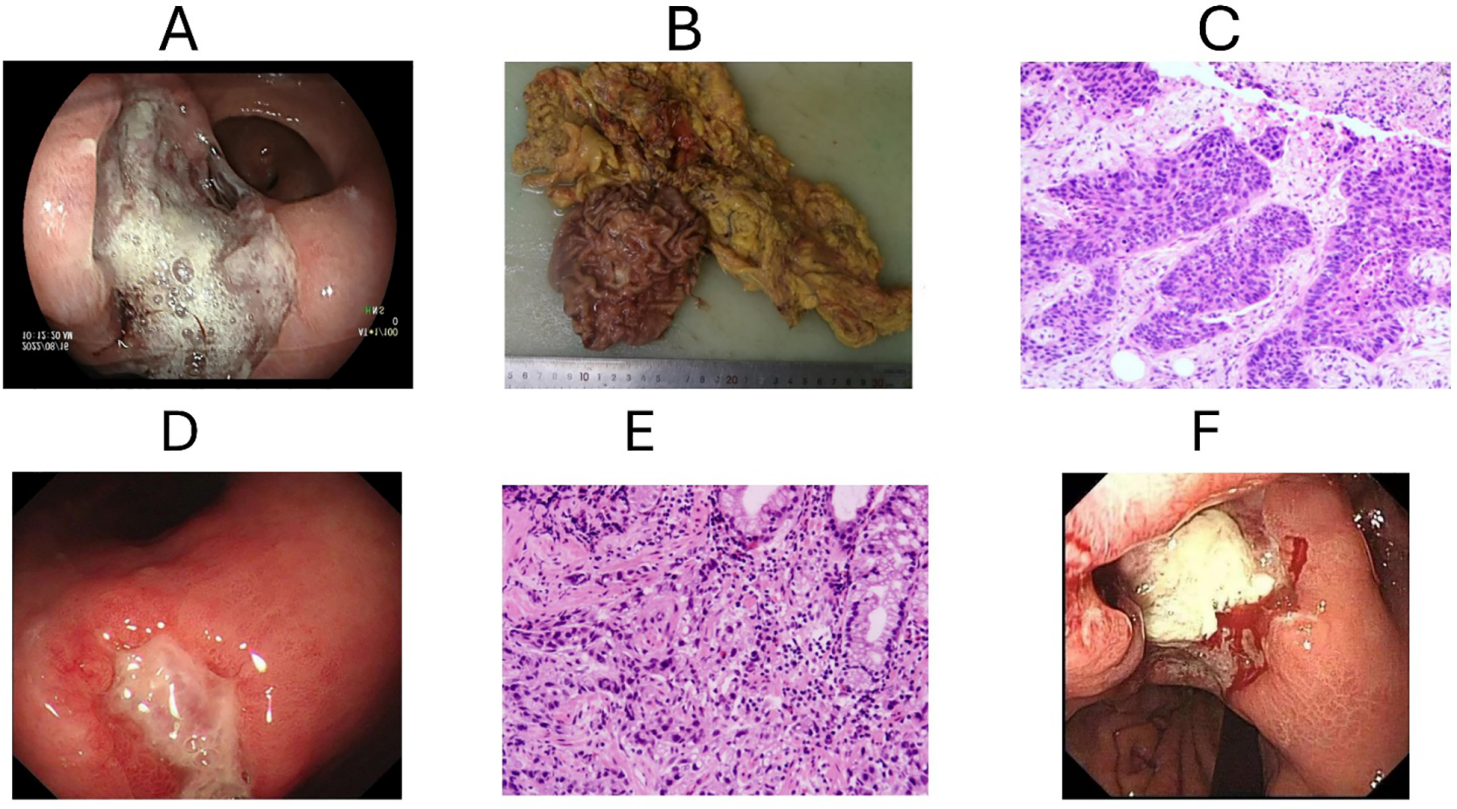
Histopathological and endoscopic evaluation. **(A)** Gastroscopy image showing ulcerated lesion in the gastric angle with white exudate and surrounding mucosal edema. **(B)** Gross specimen from radical gastrectomy showing nodular and ulcerated tumor mass in the gastric tissue. **(C)** Lung biopsy showing poorly differentiated squamous cell carcinoma (H&E stain). **(D)** Gastroscopic view showing large irregular ulcerative lesion consistent with malignancy. **(E)** Gastric biopsy revealing poorly differentiated adenocarcinoma with high-grade epithelial atypia (H&E stain). **(F)** Endoscopic image showing partially excised/bleeding lesion.

A subsequent biopsy performed on April 29, 2020, at Weihai Municipal Hospital confirmed the presence of a poorly differentiated carcinoma, consistent with squamous cell carcinoma (**Figure 2A**). Immunohistochemical analysis showed CK7 (−), TTF-1 (−), Napsin A (−), P40 (+), CK5/6 (+), and a Ki-67 proliferation index of 80%. The combined positive score (CPS) for PD-L1 was 2. The patient declined chemotherapy.

He was started on Sintilimab and Anlotinib as part of a non-cytotoxic regimen, given his refusal of chemotherapy. The response was favorable with gradual reduction in tumor burden.

On December 23, 2020, a PET-CT scan at Weihai Municipal Hospital revealed a residual hypermetabolic mass in the upper lobe of the left lung with suspected post-treatment activity. Involvement of lymph nodes in zone 4.5 and possible distant metastasis were also noted. Additionally, localized thickening of the gastric wall with increased FDG uptake was observed.

Clinically, the patient also reported an inability to eat, prompting further evaluation. Subsequent painless gastroscopy performed on December 30, 2020, at Weihai People’s Hospital revealed an ulcerative lesion measuring approximately 3.6 × 3.5 × 0.4 cm located on the lesser curvature of the gastric antrum near the gastric angle (**Figure 2B**).

Endoscopic findings reveal an ulceratic base with irregular appearance. It was covered with white exudate, surrounded by mucosal hyperemia, edema, and fragile mucosa that bled upon contact. Multiple erosions were also noted in the adjacent mucosa. The esophageal mucosa, cardia, gastric fundus, and duodenum appeared normal, while edema and congestion with mucosal thinning were observed in the gastric antrum and body. Biopsies were taken from the gastric angle (n=4) for histopathological analysis. The patient tolerated the procedure well, with stable vital signs and no immediate adverse reactions. The clinical and endoscopic diagnoses included a suspected malignant gastric ulcer (A1) and non-atrophic gastritis with erosion, warranting follow-up. Histopathology confirmed adenocarcinoma, with a PD-L1 CPS of 5. The patient remained unable to eat and once more declined surgery. Remarkably, his comorbidities, particularly diabetes mellitus, were the cause of surgical risk and recovery issues that governed his surgical refusal. Sintilimab and Anlotinib immunotherapy was therefore continued.

On November 8, 2021, follow-up endoscopy showed an enlarged ulcerative lesion (4.0 × 6.0 cm) in the gastric angle (**Figure 2D**). Contrast-enhanced CT revealed mild thickening and nodularity of the gastric wall, along with enlarged local lymph nodes. Despite disease progression, the patient continued with the initial immunotherapy regimen, refusing chemotherapy or surgery.

Due to worsening gastric obstruction, the patient underwent a radical gastrectomy at Zhujiang Hospital Southern Medical University on August 24, 2022. After surgery, the patient continued Sintilimab monotherapy.

The surgical interventions were temporally separated, with radical gastrectomy performed on August 24, 2022, followed by left upper lobectomy for lung tumor recurrence approximately fifteen months later, on November 20, 2023.

Histopathological and endoscopic evaluation revealed a moderately differentiated adenocarcinoma of the intestinal type (gastric antrum) with focal signet ring cell features (**Figures 2D, E**). The tumor invaded the muscularis propria and exhibited perineural and intravascular invasion. Lymph node metastasis was identified in 1 of 20 nodes sampled from group 9. Immunohistochemistry demonstrated positive staining for CK, CK20, CDX-2, and p53 overexpression; the Ki-67 index was approximately 90%, and PD-L1 CPS was approximately 5. HER2 was negative.

**Figure 3:**
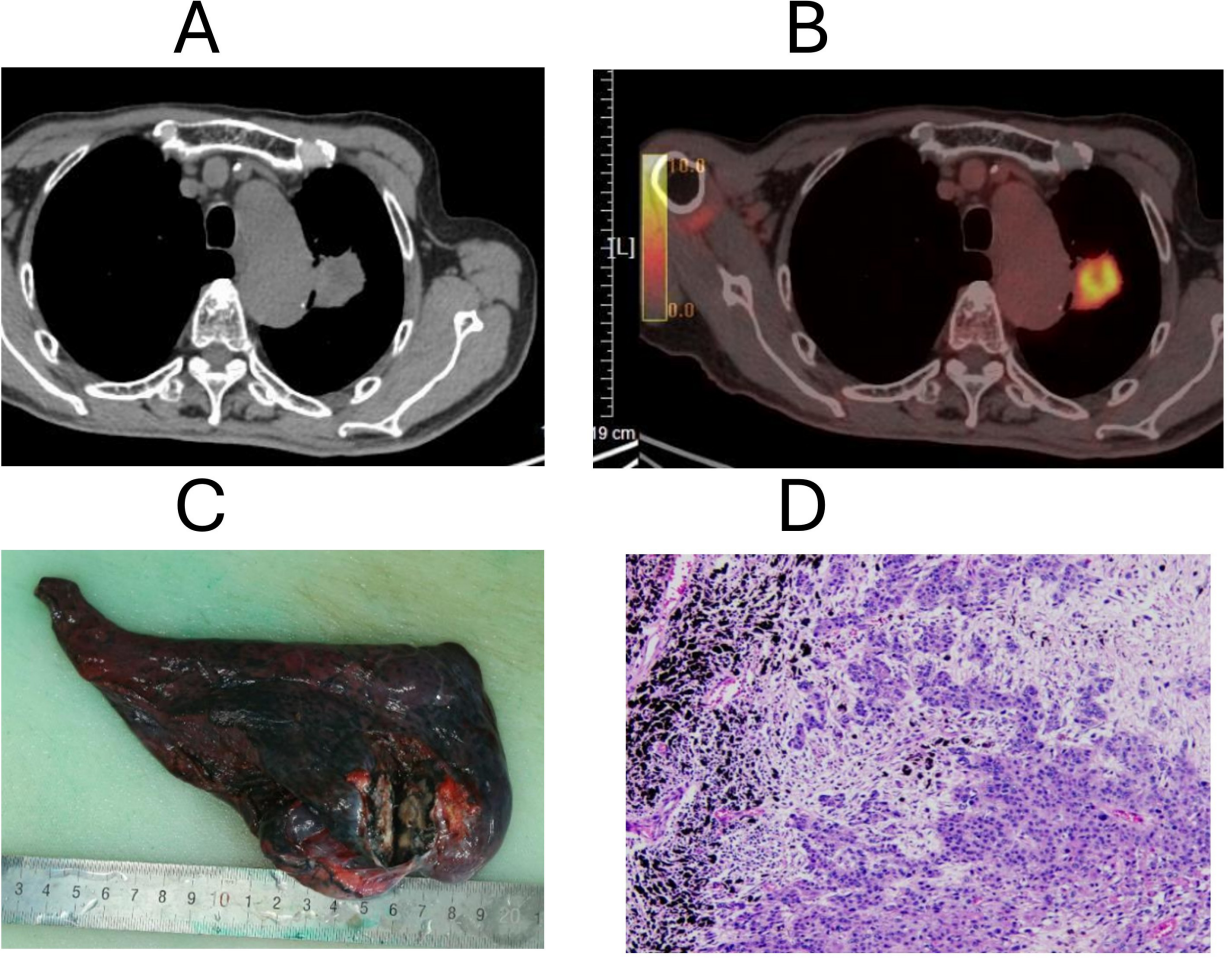
Imaging, gross pathology, and histology of lung squamous cell carcinoma. **(A)** Chest CT scan shows mass lesion in the upper lobe of the left lung. **(B)** PET-CT demonstrates increased FDG uptake at the lung lesion site. **(C)** Gross specimen of the resected left upper lobe lung tissue showing tumor mass with necrotic and ulcerated areas. **(D)** Histopathology of lung biopsy demonstrating poorly differentiated squamous cell carcinoma with keratinization (H&E stain).

In 2023, the lung lesion showed progressive enlargement. A PET-CT scan on November 7, 2023, identified a 42 × 36 mm mass in the apico-posterior segment of the left upper lobe, with irregular borders, burr-like projections, and pleural retraction. The mass showed homogeneous moderate enhancement on contrast imaging and was surrounded by newly developed small nodules with increased density (**Figure 3A**). The overall impression was local recurrence/metastasis in the left upper lobe, with recommendations for CT-guided biopsy to confirm diagnosis and continued surveillance of scattered small nodules noted in both lungs. There was no evidence of distant metastatic disease.

On November 20, 2023, the patient underwent surgical resection of the left upper lobe mass (**Figure 3B**). Histopathological examination revealed a keratinizing, moderately differentiated squamous cell carcinoma with no evidence of vascular or perineural invasion, and no alveolar or pleural spread (PL0). Resection margins were clear (**Figure 3C**). Immunohistochemical staining showed CK (+), P40 (+), P63 (+), CK5/6 (+), p53 overexpression (90%), and a Ki-67 index of ~90%. PD-L1 tumor proportion score (TPS) was approximately 2%.

**Table 1** reports the timeline and the tabulated summary of the events along with key findings, and clinical interventions.

**Table 1:** Clinical timeline of diagnosis, treatment, and surgical interventions for the patient.

Date	Event	Details
Mar 26, 2020	Chest CT: Lung lesion with metastases	Large lesion (~7.4 × 5.5 cm) in upper lobe of left lung, suspicious hilar and mediastinal lymph node involvement.
Apr 29, 2020	Lung biopsy: squamous cell carcinoma	Biopsy from upper lobe lung lesion; diagnosis: poorly differentiated squamous cell carcinoma confirmed by pathology.
Apr 2020 - ongoing	Immunotherapy started	Sintilimab 200 mg every 3 weeks + Anlotinib 8 mg daily for 14 days (21-day cycle); lesion size gradually decreased.
Jul 07 2020	followup Chest CT: lung lesion	Large lesion (~7.57× 5.69 cm) in upper lobe of left lung
Aug 06 2020	followup Chest CT: lung lesion	Large lesion (~6.25 × 5.21 cm) in upper lobe of left lung
Sep 07 2020	followup Chest CT: lung lesion	Lesion (~5.57 × 4.01 cm) in upper lobe of left lung
Oct 08 2020	followup Chest CT: lung lesion	Lesion (~4.74 × 3.15 cm) in upper lobe of left lung
Dec 28 2020	followup Chest CT: lung lesion	Lesion (~4.70 × 2.41 cm) in upper lobe of left lung
Feb 05 2021	followup Chest CT: lung lesion	Lesion (~4.45 × 2.09 cm) in upper lobe of left lung
Nov 06 2021	followup Chest CT: lung lesion	Large lesion (~4.19 × 1.88 cm) in upper lobe of left lung
Dec 09 2021	followup Chest CT: lung lesion	Lesion (~3.73 × 1.76 cm) in upper lobe of left lung
Dec 23, 2020	PET-CT: residual lung mass + gastric wall thickening	PET showed localized thickening of gastric wall with elevated FDG metabolism, suspicious for gastric lesion.
Dec 30, 2020	Gastroscopy + biopsy: gastric adenocarcinoma	Ulcerative lesion in gastric angle (~3.5 × 4.0 cm); biopsy confirmed adenocarcinoma; patient refused surgery, continued therapy.
Aug 16, 2022	Follow-up gastroscopy + biopsy	Large ulcerative lesion in gastric antrum; biopsy showed poorly differentiated adenocarcinoma with partial signet ring cell features.
Aug 24, 2022	Total gastrectomy surgery	Radical gastrectomy performed; pathology confirmed moderately differentiated gastric adenocarcinoma invading muscularis propria; lymph node metastasis present. Continued Sintilimab monotherapy post-op.
Oct 18 2023	followup Chest CT: lung lesion	Lesion (~2.79 × 2.39 cm) in upper lobe of left lung
Nov 20, 2023	Lung tumor recurrence surgery	Surgical resection of recurrent left upper lobe tumor confirmed keratinizing squamous cell carcinoma, no lymph node metastasis. Continued Sintilimab monotherapy.

Key diagnostic events include chest CT and biopsies confirming lung squamous cell carcinoma and gastric adenocarcinoma.

## 3 Diagnostic assessment and therapeutic intervention

The diagnosis of synchronous lung squamous cell carcinoma and gastric adenocarcinoma was established through cross-sectional imaging and histopathology, supported by immunohistochemical analysis including PD-L1 expression and Ki-67 proliferation indices (**Figures 1**, **2C, E**). Due to the patient’s refusal of chemotherapy and concerns related to comorbidities such as diabetes mellitus, a non-cytotoxic approach was selected. Sintilimab, a PD-1 inhibitor, was initiated alongside Anlotinib, a multi-targeted tyrosine kinase inhibitor. Following progression-related surgeries for gastric obstruction and lung recurrence (**Figures 2F**, **3C, D**), Sintilimab monotherapy was continued, resulting in sustained disease control.

## 4 Discussion

Immune checkpoint inhibitors (ICIs) have revolutionized the management of multiple solid tumors, particularly non-small cell lung cancer (NSCLC) and advanced gastric cancers. Sintilimab has emerged as a promising ICI targeting PD-1 pathway, with demonstrated clinical efficacy and manageable safety profile in both tumor types. Despite these advances, the application of ICIs in synchronous primary malignancies, particularly in case of divergent histology, including lung squamous cell carcinoma (SCC) and gastric adenocarcinoma, are not well documented and absent from major treatment guidelines.

In NSCLC, PD-1 inhibitors like Sintilimab have demonstrated substantial survival benefits, even as monotherapy in first-line settings. For instance, the ORIENT-11 trial confirmed the survival advantage of Sintilimab combined with chemotherapy in NSCLC patients, including those with squamous histology, with improved progression-free survival and acceptable toxicity (24). Meanwhile, gastric adenocarcinoma treatment has evolved to incorporate ICIs in selected populations. The ORIENT-16 trial further established the role of Sintilimab in combination with chemotherapy for unresectable or metastatic gastric or gastroesophageal junction adenocarcinoma, showing both statistically significant and clinically meaningful improvements in overall survival (25). A recent meta-analysis confirmed that Sintilimab, either alone or in combination with chemotherapy, significantly improved progression-free and overall survival in patients with advanced NSCLC. This therapeutic benefit was especially notable in patients with higher PD-L1 expression (26). In advanced squamous lung cancer, Sintilimab combined with chemotherapy showed similar efficacy and safety compared to pembrolizumab-based treatment (27).

In GCs, Sintilimab has been actively explored in both monotherapy and combination regimens. A retrospective study evaluated Sintilimab in patients with advanced or metastatic GC who had failed prior systemic therapies. The study reported a favorable response rate and a manageable safety profile, supporting Sintilimab as a viable second-line or beyond treatment option (28). In a similar line, Wei et al. (17) investigated Sintilimab combined with concurrent chemoradiotherapy in locally advanced gastric or gastroesophageal junction adenocarcinoma. Results indicated meaningful tumor downstaging with limited toxicity, suggesting a role for Sintilimab in neoadjuvant settings (17). A similar conclusion was drawn in a single-arm phase Ib trial (NCT02937116) that demonstrated high response rates and promising survival outcomes (16). Furthermore, Sintilimab and chemotherapeutic agents [e.g., nab-paclitaxel and S-1] have also been studied widely (29, 30).

In this case, Sintilimab was initially combined with Anlotinib, an oral multi-targeted tyrosine kinase inhibitor. Anlotinib inhibits a range of signaling pathways involved in tumor angiogenesis and growth, including VEGFR 1/2/3, FGFR 1–4, PDGFR α/β, c-Kit, and RET. This combination may have exerted synergistic effects: Anlotinib’s anti-angiogenic and antiproliferative action likely disrupted the tumor microenvironment, facilitating immune activation via PD-1 blockade. Although the patient was later managed with Sintilimab monotherapy, the initial tumor regression observed could partially be attributed to this dual-targeted approach. Such combinations may be particularly valuable in cases where standard chemotherapy is not an option.

Despite this growing body of evidence for single-agent and combination ICI use, treatment guidance remains limited for patients with synchronous cancers, due to the rarity of such presentations and the absence of dedicated clinical trials. In their 2024 case study, Ghammem et al. highlight this gap, reporting that guideline-directed monotherapy with ICIs is uncommon in such complex scenarios due to limited clinical evidence and concerns about differential tumor immune microenvironments (21).

The case report presents a rare clinical case of synchronous lung SCC and gastric adenocarcinoma, comprising histological diagnosis and successful treatment with a single-agent immune checkpoint inhibitor (ICI), Sintilimab. The patient, a 64-year-old male, initially presented with an irregular upper lobe left lung mass on chest CT. Subsequent diagnostic workup established poorly differentiated lung SCC with extensive lymph node metastasis. A gastric lesion was later incidentally discovered because of clinical presentation, and histopathology revealed adenocarcinoma with focal signet ring cell differentiation.

A detailed family history was obtained and did not reveal evidence of hereditary cancer syndromes or early-onset malignancies among first-degree relatives. Moreover, the relatively late age of cancer onset at 64 years further reduces the likelihood of an inherited multicancer syndrome, such as Li-Fraumeni syndrome, which is typically characterized by early-onset malignancies associated with germline TP53 mutations.

The clinical course in this patient was particularly noteworthy in that both tumors were successfully managed with Sintilimab monotherapy, despite the patient’s constant refusal of chemotherapy and initial resistance to surgery. The patient’s long-standing diabetes mellitus was most likely responsible for his reluctance to accept invasive therapies like surgery, in the context of the risk of complications like perioperative morbidity, infection, and wound healing delay. This underlines the importance of individualized oncologic therapy to consider comorbidities, which may determine both the eligibility for treatment and patient acceptability. As discussed above, Sintilimab, a monoclonal anti-PD-1 antibody, has activity in a broad spectrum of cancer types, including NSCLC and GC. Effective monotherapy in synchronous, histologically different tumors, however, is very rare and the case thus contributes significantly to literature.

Pathological and immunohistochemical examination provided substantial evidence supporting continuous immunotherapy. Both the tumors also showed high proliferative activity, as indicated by high Ki-67 indices (around 80% in lung SCC and 90% in gastric adenocarcinoma), reflecting aggressive behavior of the tumor. Both the cancers also showed positive PD-L1 expression (Combined Positive Score [CPS] of 2 in lung SCC and 5 in gastric adenocarcinoma), which rationalize the observed efficacy with anti-PD-1 therapy.

Most importantly, Sintilimab treatment achieved longer than three years of sustained disease control. The patient initially received Sintilimab combined with Anlotinib and was subsequently switched to Sintilimab monotherapy following surgical resection of gastric tumor. The response to immunotherapy was consistently favorable with partial imaging responses and improvement in clinical symptoms. Finally, the gastric adenocarcinoma required surgical resection of worsening gastric obstruction and therefore underwent radical gastrectomy. Following pathological review, there was complete resection with minimal nodal disease and no residual disease. Similarly, following progressive enlargement of the lung lesion, the patient underwent a left upper lobectomy for recurrent squamous cell carcinoma. Histopathological examination demonstrated clear surgical margins with no residual invasive or metastatic disease.

The tolerance of the patient to immunotherapy and long-term postoperative outcomes highlight therapeutic potential and clinical feasibility of employing ICI monotherapy in multiple synchronous cancers, albeit selected due to lack of extensive clinical evidence.

With the complicated clinical scenario, the case highlights the therapeutic potential of immunotherapy as an alternative, especially for those who cannot or do not wish to receive conventional chemotherapy.

From a wider clinical context, this report supports the concept of patient-specific, individualized care regimens. In the absence of universal guidelines for synchronous cancers treated with ICIs alone, this positive outcome provides clinical evidence, though limited, in favor of such treatments. Existing literature primarily supports multimodal regimens with chemotherapy and surgery, which further establishes the novelty of employing single-agent ICIs in this context. While this single case cannot define generalized treatment regimens, it provides robust clinical evidence in favor of investigating immunotherapy as monotherapeutic agents for synchronous tumors.

Nonetheless, the case report does acknowledge certain limitations. The patient’s refusal of conventional chemotherapy might have yielded limited insight into how combinational approaches might have influenced outcomes. Second, the prolonged delay in surgery might have permitted progression of the disease and risk of metastasis, potentially making clinical management more difficult. Despite these limitations, the success achieved by Sintilimab monotherapy is remarkable and clinically informative.

Additional research, ideally prospective and multicenter, is required to establish definitively the broader applicability and efficacy of ICIs for synchronous primary cancer therapy. Molecular and immunology profiling with specific focus could further refine patient selection criteria to better achieve therapeutic effects. An ultimate research goal should also be to establish mechanisms of differential response among synchronous tumors to further maximize therapeutic individualization.

In summary, this case describes a rare but successful clinical outcome in synchronous SCC of the lung and gastric adenocarcinoma concurrently treated with Sintilimab monotherapy. The durable disease control, patient tolerability, and favorable postoperative stability demonstrate the therapeutic potential of ICIs in complicated oncologic presentations. Lastly, these results demonstrate the therapeutic potential of immunotherapy regimens tailored to the individual to revolutionize management paradigms in the patient with synchronous, dissimilar malignancies.

## 5 Patient perspective

The patient expressed gratitude for the care received and reported no complications from the treatment. He remains generally healthy and satisfied with the outcome of the therapy.

## Data Availability

The original contributions presented in the study are included in the article/supplementary material. Further inquiries can be directed to the corresponding author.
